# Synbiotic regulates gut microbiota in patients with lupus nephritis: an analysis using metagenomic and metabolome sequencing

**DOI:** 10.3389/fmicb.2024.1295378

**Published:** 2024-04-02

**Authors:** Qiuyu Zhu, Jiuming Cui, Sen Liu, Suosu Wei, Qiuxia Wu, Yanwu You

**Affiliations:** ^1^Department of Nephrology, Affiliated Hospital of Youjiang Medical University for Nationalities, Baise, China; ^2^Department of Scientific Cooperation, Guangxi Academy of Medical Sciences, People’s Hospital of Guangxi Zhuang Autonomous Region, Nanning, China; ^3^Department of Nephrology, People’s Hospital of Guangxi Zhuang Autonomous Region, Nanning, China

**Keywords:** lupus nephritis, gut microbiota, synbiotics, metagenomics, metabolomics

## Abstract

**Objective:**

To investigate the changes in gut microbes and their metabolites after administering synbiotics to patients with new-onset lupus nephritis (LN) treated using a conventional method and provide a theoretical basis for finding new targets for the diagnosis and treatment of LN.

**Methods:**

In this study, a total of 12 participants were divided into the lupus and synbiotic groups. Stool samples and clinical data were collected before and after treatment for metagenomic, nontargeted metabolomic, and statistical analyses.

**Results:**

The relative abundances of the pathogenic bacteria *Prevotella, Bacteroides,* and Enterobacteriaceae_unclassified decreased after synbiotic treatment, whereas the abundances of Actinobacteria and Firmicutes increased. Further, the Firmicutes to Bacteroidetes ratio increased; however, the difference was not statistically significant (*p* > 0.05). α diversity analysis showed no significant differences in the intestinal microbial richness and diversity index of patients with LN between the groups before and after treatment (*p* > 0.05). β analysis showed the differences in the community structure between the samples of the two groups before and after treatment. Linear discriminant analysis effect size and receiver operating characteristic curve analyses revealed that Negativicutes (AUC = 0.9722) and Enterobacteriaceae_unclassified (AUC = 0.9722) were the best predictors of the lupus and synbiotic groups, respectively, before and after treatment. Joint analyses revealed that amino acid biosynthesis, aminoacyl-tRNA biosynthesis, purine metabolism, and other metabolic pathways may be involved in the changes in the metabolic function of patients with LN after the addition of synbiotics. Spearman’s correlation analysis revealed the interaction between clinical features and flora, and flora exhibited a complex biological network regulatory relationship.

**Conclusion:**

Synbiotics regulate the metabolic functions of intestinal microorganisms in patients with LN and play a role in various biological functions. Synbiotic supplements may be safe and promising candidates for patients with LN.

## 1 Introduction

Systemic lupus erythematosus (SLE) is a chronic autoimmune disease with varying severity of multisystem and multiorgan damage. Lupus nephritis (LN) is a serious SLE complication that physically and mentally burdens both patients and society. Health burden (Almaani et al., 2017; Yuan et al., 2019) and SLE pathogenesis are closely related to genetics and the environment; however, the etiology is currently unknown. Notably, disturbances in the intestinal flora may be involved in SLE pathogenesis (Sonnenburg and Bäckhed, 2016). Environmental and biological factors can trigger the development of lupus, but the role of gut microbes in its development has not been clarified; therefore, clarifying the interaction between LN and the gut flora is the focus of current research. The intestinal microecology of patients with lupus can be adjusted through diet, probiotics, and prebiotics, along with the restoration of the microecological balance, ultimately enhancing the therapeutic effect. Therefore, identifying the exact intestinal microbes leading to the deterioration of LN symptoms and further understanding the intestinal microbiota are imperative. Changes in the characteristics of group metabolomics may help reveal the relationship between intestinal flora and host health and disease and identify lupus treatment methods targeting intestinal microecology (Adak and Khan, 2019; Zheng et al., 2020). The World Health Organization defines probiotics as live microorganisms that confer health benefits to the host when administered in sufficient quantities (Makharia et al., 2022). Probiotics or prebiotics are safe and effective in modulating intestinal flora (Plaza-Diaz et al., 2019). Notably, synbiotics (probiotics and prebiotics) have better efficacy than probiotics and prebiotics alone, and using probiotics and synbiotics is a promising strategy to regulate the intestinal microbiota (Santos-Cruz et al., 2022). In this study, using gut microbes and their metabolites as the starting point, we observed changes in the gut microbes and their metabolites after administering synbiotics to patients with newly diagnosed LN treated using a conventional method through metagenomic sequencing and non-targeted metabolome sequencing analysis. Further, we explored the mechanism by which intestinal flora participates in the occurrence and development of LN after the addition of synbiotics to provide a theoretical basis and novel insights for identifying new targets for LN diagnosis and treatment.

## 2 Materials and methods

### 2.1 General information

This study recruited 12 newly diagnosed patients with LN from the Nephrology Department of the Affiliated Hospital of Youjiang National Medical College from September 2020 to August 2021. All patients were female, and most were from Zhuang. All patients were from southern China with similar geographical regions and eating habits and exhibited no specific dietary patterns. No significant differences existed in age, weight, or BMI (*p* > 0.05), as shown in Table 1. All patients with SLE met the SLE classification criteria revised by the American Rheumatism Association criteria (ARA) in 1997 (Hochberg, 1997). Patients with LN also met the above SLE classification criteria and exhibited >1 of the following manifestations: (1) proteinuria: >0.5 g/24 h; (2) casts: red blood cells, hemoglobin (HB), granular casts, or mixed casts; and (3) renal biopsy: confirmed LN, and the pathological changes conformed to the International Society of Nephrology/Society of Renal Pathology classification criteria. This study was approved by the Ethics Committee of the Affiliated Hospital of Youjiang Medical University for Nationalities (YYFY-LL-2020-031). All participants provided informed consent prior to the experiment.

**Table 1:** Basic information of the two groups of patients.

Variable	Lupus group (*n* = 6)	Synbiotic group (*n* = 6)	*P* value
Age	29.16 ± 6.33	27.71 ± 6.44	0.691
Weight (Kg)	48.48 ± 5.91	51.53 ± 7.32	0.446
BMI (Kg/m^2^)	19.88 ± 3.37	23.18 ± 4.48	0.159

The data were unpaired *T*-test. *p* < 0.01 means the difference is very significant, 0.01 < *p* < 0.05 means the difference is significant, *p* > 0.05 means the difference is not significant.

#### 2.1.1 Inclusion criteria

The patients were recruited according to the following inclusion criteria: (1) Conforming to the ARA diagnostic criteria for SLE 1997; (2) Age 18–60 years; (3) No overlap with other rheumatic diseases such as rheumatoid arthritis and Sjogren’s syndrome; (4) Before sampling, the participants had not received antibiotics, probiotics, prebiotics, or other foods and drugs that regulate intestinal flora for at least 6 months; (5) All patients were from southern China, with similar geographical regions and eating habits and no specific dietary patterns.

#### 2.1.2 Exclusion criteria

Patients (1) with other autoimmune diseases, primary glomerulopathy, tumor, epilepsy, organic encephalopathy, mental illness, idiopathic thrombocytopenic purpura, and other diseases; (2) with respiratory and digestive tract infections; (3) admitted to the hospital and had previously taken glucocorticoids or immunosuppressant drugs; and (4) who lost follow-up were excluded from the study.

### 2.2 Methods

#### 2.2.1 Stool sample and clinical data collection

Twelve patients with newly diagnosed LN were randomly divided into the lupus (*n* = 6) and synbiotic (*n* = 6) groups. The lupus and synbiotic groups were treated with prednisone acetate 1.0 mg/kg/d and cyclophosphamide. In addition, the synbiotic group was administered synbiotics (Shanghai Jiaoda Only Co Ltd., China) at a dosage of 5 × 10^10^ colony-forming units/day. This synbiotic formulation contains eight active strains—*Lactobacillus rhamnosus*, *L. acidophilus*, *L. plantarum*, *L. lacidobacterium*, *L. paracei*, *L. casei*, *Bifidobacterium brevis*, and *B. longum*. In addition, it contains prebiotic ingredients: fructooligosaccharide, erythritol, maltodextrin, xylooligosaccharide, and orange fruit powder. The stool samples of the two groups before and after 30 days of treatment were collected and frozen in the laboratory at −80°C in a refrigerator for backup. Subsequently, all samples were transported on dry ice to Hangzhou Lianchuan Biotechnology Co. Ltd. for sequencing analysis. Clinical data from the two groups were collected before and after 30 days of treatment.

#### 2.2.2 Metagenome sequencing experiments and bioinformatics analysis

Bacterial DNA was extracted using the EZNA®Stool DNA Kit (D4015, Omega Inc., USA) following the manufacturer’s instructions, and the genomic DNA was used for the library construction by LC-Bio Technologies Co. Ltd., Hangzhou, China. A DNA library was constructed using a TruSeq Nano DNA LT Library Preparation Kit (FC-121-4001). Raw sequencing reads were processed to obtain valid reads for further analyses. Sequencing adapters were removed from the sequencing reads using Cutadapt v1.9. Subsequently, low-quality reads were trimmed using fqtrim v0.94, a sliding window algorithm. The reads were aligned to the host genome using Bowtie2 v2.2.0 to remove host contamination. After obtaining the quality-filtered reads, they were assembled *de novo* to construct the metagenome for each sample using IDBA-UD v1.1.1. All coding regions (CDS) of the metagenomic contigs were predicted using MetaGeneMark v3.26. The CDS sequences of all samples were clustered using CD-HIT v4.6.1 to obtain the unigenes. The unigene abundance in each sample was estimated by TPM based on the number of aligned reads using Bowtie2 v2.2.0. The lowest common ancestor taxonomy of the unigenes was obtained by aligning them against the NCBI NR database using Diamond v0.9.14. Similarly, functional annotations (Gene Ontology, Kyoto Encyclopedia of Genes and Genomes [KEGG], eggnog, CAZy, CARD, PHI, MGEs, and VFDB) of the unigenes were obtained. Based on the taxonomic and functional annotation of unigenes and the abundance profile of unigenes, differential analysis was performed at the taxonomic, functional, or gene level using Fisher’s exact (non-replicated groups) or Kruskal–Wallis (replicated groups) tests.

#### 2.2.3 Non-targeted metabolome experiments and bioinformatics analysis

Metabolites were extracted from fecal and serum samples using 50% methanol buffer and incubated at 24°C for 10 min. The extracted mixture was centrifuged at 4000 × g for 20 min, and the supernatant was used for liquid chromatography–mass spectroscopy (LC–MS) analysis to identify the metabolites. Chromatographic separations were performed using an ultra-performance liquid chromatography (UPLC) system (SCIEX, UK) and ACQUITY UPLC HSS T3 column (100 × 2.1 mm, 1.8 μm, Waters, UK). A high-resolution tandem mass spectrometer (TripleTOF 5,600 Plus, SCIEX, UK) was used for both PIM and NIM to detect the metabolites eluted from the column. The Q-TOF mass range was 60–1,200 Da. XCMS software was used to pretreat the acquired MS data. The raw LC–MS data files were processed using MetaX with the XCMS package for peak detection and the CAMERA package for peak annotation, all based on R. Each ion was identified by combining the retention time and m/z data. The KEGG and Human Metabolome Database (HMDB) were used to annotate the metabolites using the exact molecular mass data (m/z) of the samples. Student’s t-tests were used to detect differences in metabolite concentrations between the two groups. The *p*-value was adjusted for multiple tests using the false discovery rate (Benjamini–Hochberg).

### 2.3 Statistical processing

Statistical analysis was performed using SPSS 25.0, and data are expressed as the mean, standard deviation (for data with a normal distribution), and median (interquartile range for data with a skewed distribution). Normally distributed data were analyzed using paired or independent t-tests; otherwise, nonparametric Wilcoxon rank-sum tests and Mann–Whitney U tests were used for comparisons. Correlation analysis was performed using Spearman’s test, and statistical significance was set at *p* < 0.05.

## 3 Results

### 3.1 Clinical data and laboratory test results

The datas were analyzed for variance, and the indicators of homogeneity of variance (*p* > 0.05) include WBC, C3, CH, IgG, and SLEDAI; For homogeneity of variance, LSD-t test is used for multiple comparisons; otherwise, Tamheini test is used for multiple comparisons. The results are expressed as mean and 95% CI.*: after multiple tests and corrections, the *p*-value<0.0083 is significant because we must correct the analysis of multiple tests (the *p*-value of 0.0083 is calculated as 0.05 multiplied by 6). The results showed that the SLEDAI, 24-h urine protein, WBC and other indicators decreased before and after treatment in both groups, while the ALB, HB, GFR and other indicators increased. SLEDAI showed statistical differences between the two groups after treatment (*p* < 0.01), while complement C4, ALB, and GFR showed statistical differences before and after treatment in the lupus group (*p* < 0.01) (Table 2).

**Table 2:** Comparison of clinical indexes before and after treatment in lupus group and synbiotic group.

Group index	LNA	LNPA	LNB	LNPB	*P* value		
					LNA compare with LNB	LNPA compare with LNPB	LNB compare with LNPB
WBC (10^9/L)	6.09(4.28–7.89)	9.11(7.39–10.82)	6.45(4.51–8.39)	6.91(5.11–8.72)	0.721	0.044	0.667
HB (g/L)	85.16(55.39–114.93)	110.28(83.04–137.52)	132.00(123.37–140.62)	130.60(122.06–139.13)	0.05	0.544	1
PLT (10^9/L)	261.00(179.62–342.37)	327.14(237.51–416.76)	307.33(274.45–340.21)	358.60(217.88–499.31)	0.774	0.997	0.941
ALB (g/L)	29.70(25.35–34.04)	35.58(27.42–43.74)	59.73(47.24–72.22)	56.12(43.73–68.51)	0.006^*^	0.036	0.996
GFR (ml/min)	51.62(33.97–69.27)	73.61(38.46–108.75)	107.74(99.84–115.64)	117.92(108.93–126.90)	0.001^*^	0.12	0.262
TG (mmol/L)	1.88(1.43–2.34)	1.32(0.61–2.02)	1.44(1.09–1.78)	0.85(40.34–1.36)	0.38	0.744	0.181
SLEDAI (score)	9.16(6.64–11.68)	5.14(3.04–7.24)	7.50(5.65–9.34)	3.2(1.83–4.56)	0.164	0.113	0.002^*^
24 h-Upro (g/L)	1.76(0.82–2.71)	2.89(0.32–5.45)	1.40(0.61–2.18)	1.16(0.46–2.77)	0.976	0.701	1
Scr (umol/L)	97.16(30.88–163.44)	71.71(46.03–97.39)	60.83(56.77–64.89)	56.60(46.64–66.55)	0.772	0.763	0.905
CH (mmol/L)	4.35(3.38–5.32)	5.30(3.93–6.67)	5.03(4.06–6.00)	4.01(2.90–75.12)	0.299	0.062	0.146
C3 (g/L)	0.59(0.41–0.76)	0.81(0.61–1.01)	0.87(0.69–1.05)	1.02(0.85–1.18)	0.013	0.065	0.198
C4 (g/L)	0.08(0.04–.12)	0.25(0.04–0.45)	0.75(0.57–0.92)	0.55(0.23–0.87)	0.001^*^	0.338	0.714
IgG (g/L)	28.92(13.61–44.23)	18.02(5.31–30.73)	13.88(9.98–17.78)	15.19(12.17–18.20)	0.024	0.656	0.842

WBC, White Blood Cell; HB, Hemoglobin; PLT, Platelet; ALB, Plasma Albumin; GFR, Glomerular Filtration Rate; TG, Triglycerides; SLEDAI, SystemicLupus Erythematosus Disease Activity Index; 24 h-UPro, 24 h Urine Protein; Scr, Serum Creatinine; CH, Cholesterol; C3, Complement C3; C4, Complement C4; IgG, Immunoglobulin G. LNA, lupus group before treatment; LNB, lupus group after treatment; LNPA, synbiotic group before treatment; LNPB, synbiotic group after treatment. *After multiple tests and corrections, the *p*-value<0.0083 is significant.

### 3.2 Analysis of the effect of synbiotics on the intestinal flora of patients with LN based on metagenomic sequencing

**Figure 1:**
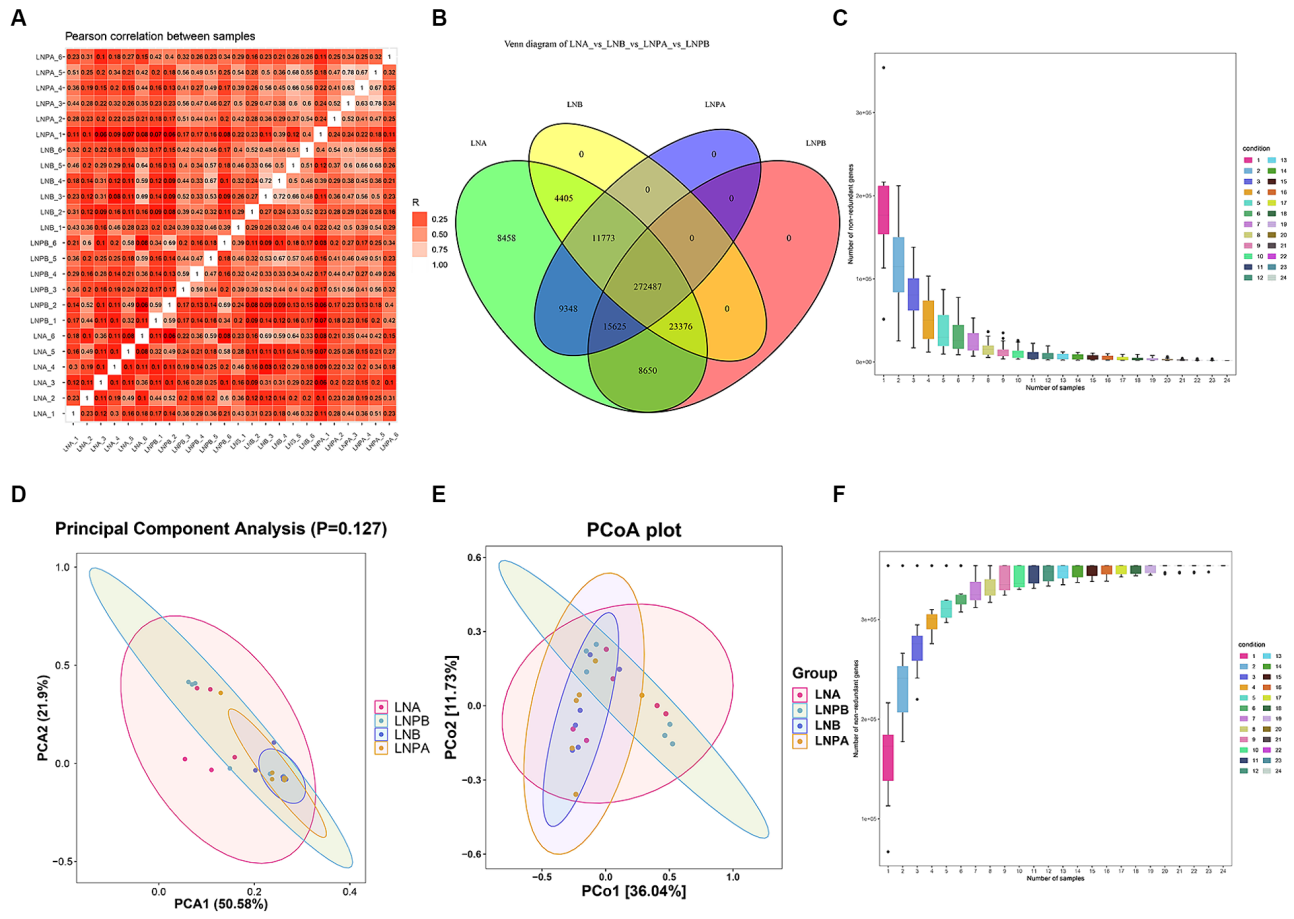
Correlation of all samples, number of genes, and β diversity analysis. **(A)** Sample correlation analysis based on gene number, where different colors represent high or low correlation coefficients. The relationship between the correlation coefficient and color is shown in the legend on the right; the darker the color, the greater the absolute value of the correlation coefficient between samples. **(B)** Gene number Venn diagram analysis showing the numbers in the circles and overlapping parts representing the number of genes shared between samples, and the numbers without overlapping parts representing the number of unique genes in the sample. **(C)** Number of gene intersections between sample combinations in the core curve. **(D)** β diversity PCA^a^ analysis. **(E)** β diversity principal coordinate analysis. **(F)** Number of gene unions between sample combinations in the core curve. ^a^Principal component analysis.

To understand the changes in the intestinal flora of patients with LN after the administration of synbiotics, a correlation analysis was performed on all samples. The results showed a good correlation between samples and repeatability within the group, and the difference between groups was acceptable (Figure 1A). The Venn diagram (Figure 1B) revealed 272,487 unigenes in the four groups: 8,458 and 9,348 unigenes before treatment in the lupus and synbiotic groups, respectively, and 312,041 and 288,112 unigenes before and after treatment in the lupus and synbiotic groups, respectively. The core/pan curve revealed the number of common and unique genes in all samples, and the plateau curve indicated that the sequencing sample size was reasonable and met the experimental standards (Figures 1C,F). To assess the degree of similarity between microbial communities, an intestinal microbial β diversity analysis of patients with LN after administering synbiotics was performed. The results indicated certain commonalities and uniqueness in the composition of intestinal flora between the two groups (Figures 1D,E).

**Figure 2:**
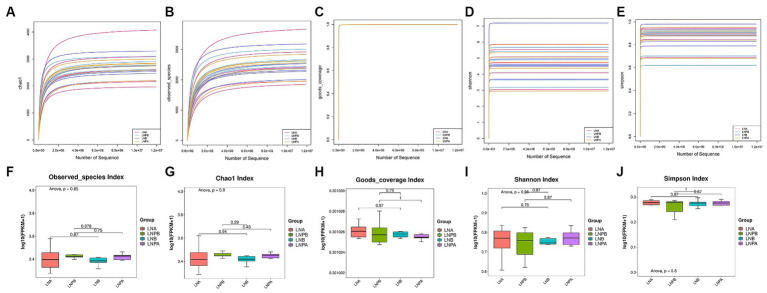
α diversity analysis and box plot comparison of the intestinal microbiota before and after treatment in the two groups. **(A)** α diversity analysis using the Chao1 index. **(B)** α diversity analysis of the specification index. **(C)** α diversity analysis of the Goods coverage index. **(D)** α diversity analysis of the Shannon Index. **(E)** α diversity analysis of the Simpson’s index. **(F)** Specifications index box chart. **(G)** Chao1 index box chart. **(H)** Goods coverage index box chart. **(I)** Shannon index box plot. **(J)** Simpson’s index box plot.

We performed α diversity analysis on fecal samples collected before and after treatment from the two groups to evaluate the richness and diversity of the microbial communities within the samples. The flatness of the sparse curve indicated that the sequencing data were sufficient to cover all bacterial species in the community, confirming that the amount of sequencing data for the samples collected in this study was sufficient (Figures 2A–E). However, the Chao1, Observed_species, Goods_coverage, and Shannon and Simpson indices were not significantly different between the two groups before and after treatment (*p* > 0.05) (Figures 2F–J).

**Figure 3:**
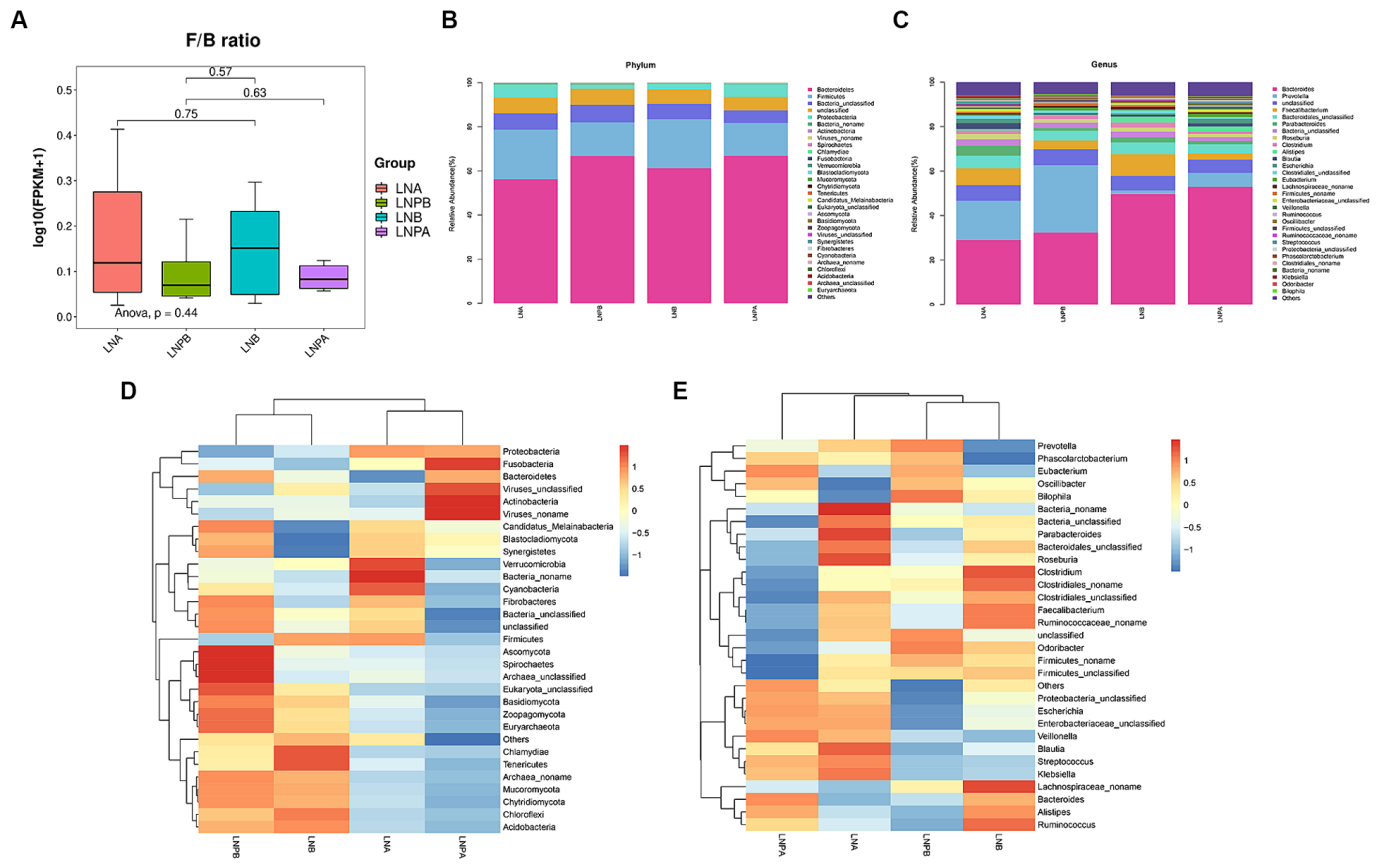
Composition and clustering heatmap of the gut microbiota in the top 30 relative contents. LNA, lupus group before treatment. LNB, lupus group after treatment. LNPA, synbiotic group before treatment. LNPB, synbiotic group after treatment. **(A)** Comparison of the F/B ratios between the two groups before and after treatment. **(B)** The top 30 most abundant microorganisms at the phylum level. **(C)** The top 30 relatively abundant microorganisms at the genus level. **(D)** Gate-level clustering heatmap. **(E)** Genus-level clustering heatmap.

Based on the sample abundance table, we can test for species differences between samples or groups of samples. Difference test methods are Fisher’s exact test and Mann Whitney U test, in which Fisher’s exact test is suitable for difference comparison of samples without biological duplication. The Mann Whitney U test was used to compare the difference between two groups of samples with biological replication. At the phylum level, the intestinal colony structure of patients with LN mainly comprised of Bacteroidetes, Firmicutes, Bacteria_unclassified, Proteobacteria, and Actinobacteria. In the lupus group, the abundance of Bacteroidetes and Firmicutes after treatment increased and decreased, respectively after treatment. After treatment with synbiotics, the abundance of Firmicutes increased, implying an increase in the Firmicutes to Bacteroidetes (F/B) ratio; however, the difference was not statistically significant (*p* > 0.05) (Figure 3A). The relative abundance of Proteobacteria decreased significantly after treatment in the two groups compared with that before treatment. A statistically significant difference was also observed before and after treatment in the synbiotic group (*p* = 0.02, *p* < 0.05), and the expression of Proteobacteria was upregulated. Actinobacteria showed a statistically significant difference before and after treatment in the synbiotic group (*p* = 0.042, *p* < 0.05), and its expression was downregulated (Figure 3B). These results indicated an imbalance in the gut microbiota in patients with LN. The genera mainly comprised *Bacteroides*, *Prevotella*, *Faecalibacterium*, and *Parabacteroides*. The abundance of *Prevotella* significantly decreased before and after treatment in the lupus group (*p* = 0.04, *p* < 0.05) and its expression was upregulated (Figure 3C). To further observe the differences in community composition between the two groups before and after treatment, hierarchical clustering and heat map analyses were performed on the top 30 species and samples with the highest abundances (Figures 3D,E).

**Figure 4:**
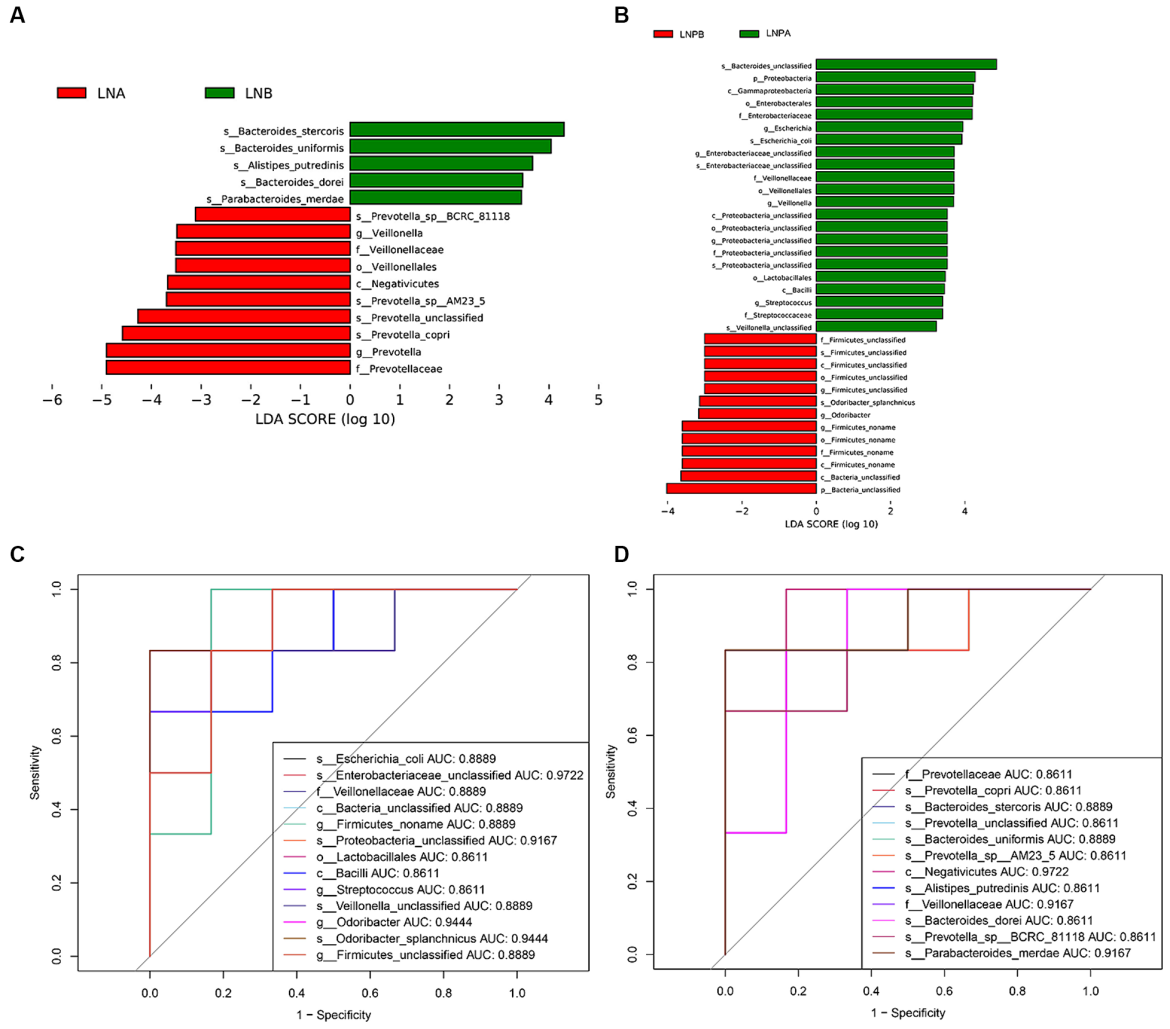
Differential analysis of gut microbiota before and after treatment in two groups. **(A)** Histogram of LDA^a^ value distribution before and after treatment in the lupus group. **(B)** Histogram of LDA value distribution before and after treatment in the synbiotic group. **(C)** ROC^b^ curve analysis of differential microbiota in the lupus group before and after treatment. **(D)** ROC curve analysis of differential microbiota in the synbiotic group before and after treatment. ^a^Linear discriminant analysis; ^b^Receiver operating characteristic curve.

Kruskal-Wallis rank sum test was used to detect all characteristic species, and significantly different species were obtained by detecting species abundance differences among different groups. Then the Wilcoxon rank sum test was used to detect whether all subspecies of the significantly different species obtained in the previous step converged to the same taxonomic rank. Then linear discriminant analysis (LDA) was used to obtain the final differential species (biomarker). Linear discriminant analysis (LDA) effect sizes (LEfSe) were used to identify the characteristic microbiota (*p* < 0.05), with different characterisations performed using an LDA cutoff value of 3.0. Fifteen biomarkers were identified in the lupus group before and after treatment, among which Prevotellaceae showed the highest score (LDA = 4.90). A comparison of the 35 biomarkers before and after treatment in the synbiotic group revealed that *Bacteroides*_unclassified exhibited the highest score (LDA = 4.84) (Figures 4A,B). To explore the predictive ability of potential biomarkers to distinguish LN status according to the above results, we used the receiver operating characteristic curve (ROC) to evaluate the predictive performance of the screened biomarkers and select the best predictive performance model (Figures 4C,D). Enterobacteriaceae_unclassified and Negativicutes were the best predictors before and after treatment for the synbiotic (AUC = 0.9722) and lupus (AUC = 0.9722) groups, respectively.

**Figure 5:**
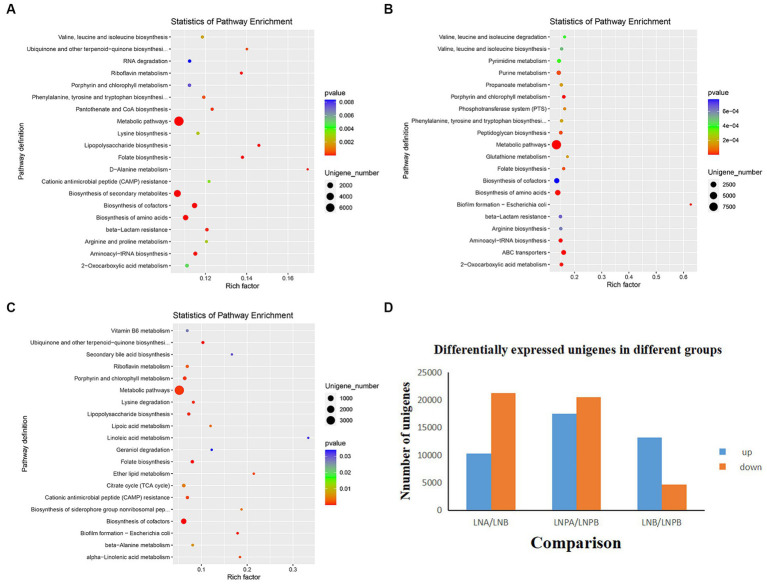
Histogram and KEGG enrichment analyses of differentially expressed genes. **(A)** KEGG^a^ enrichment analysis in the lupus group before and after treatment. **(B)** KEGG enrichment analysis before and after synbiotic treatment. **(C)** KEGG enrichment analysis after treatment in the lupus and synbiotic groups. **(D)** Histograms of differentially expressed genes before and after treatment in both groups (each point in the figure represents a pathway; the size and color depth of the points represent the correlation with the influence of the pathway, which is the ratio of the number of differentially expressed genes in the pathway to the total number of genes). ^a^Kyoto Encyclopedia of Genes and Genomes.

To determine how synbiotics act on the intestinal flora of patients with LN, leading to differential changes at the gene level, we analyzed all genomic information of the intestinal flora (Figure 5D). KEGG enrichment analysis revealed 140 enriched pathways in the lupus group before and after treatment, among which 35 were significantly differentially enriched (*p* < 0.05), mainly in the biosynthesis of secondary metabolites, amino acids, and cofactors. Further, 149 enriched pathways were identified before and after treatment in the synbiotic group, among which 44 were significantly differentially enriched (*p* < 0.05), mainly in the biosynthesis of amino acids, cofactors, and ABC transport. Notably, 139 enriched pathways were identified in the lupus group compared with those in the synbiotic group after treatment, of which 22 were significantly enriched (*p* < 0.05), mainly in cofactor biosynthesis, porphyrin and chlorophyll metabolism, and folic acid biosynthesis (Figures 5A–C).

### 3.3 Effect of synbiotics on the intestinal flora of patients with LN based on non-targeted metabolome sequencing

**Figure 6:**
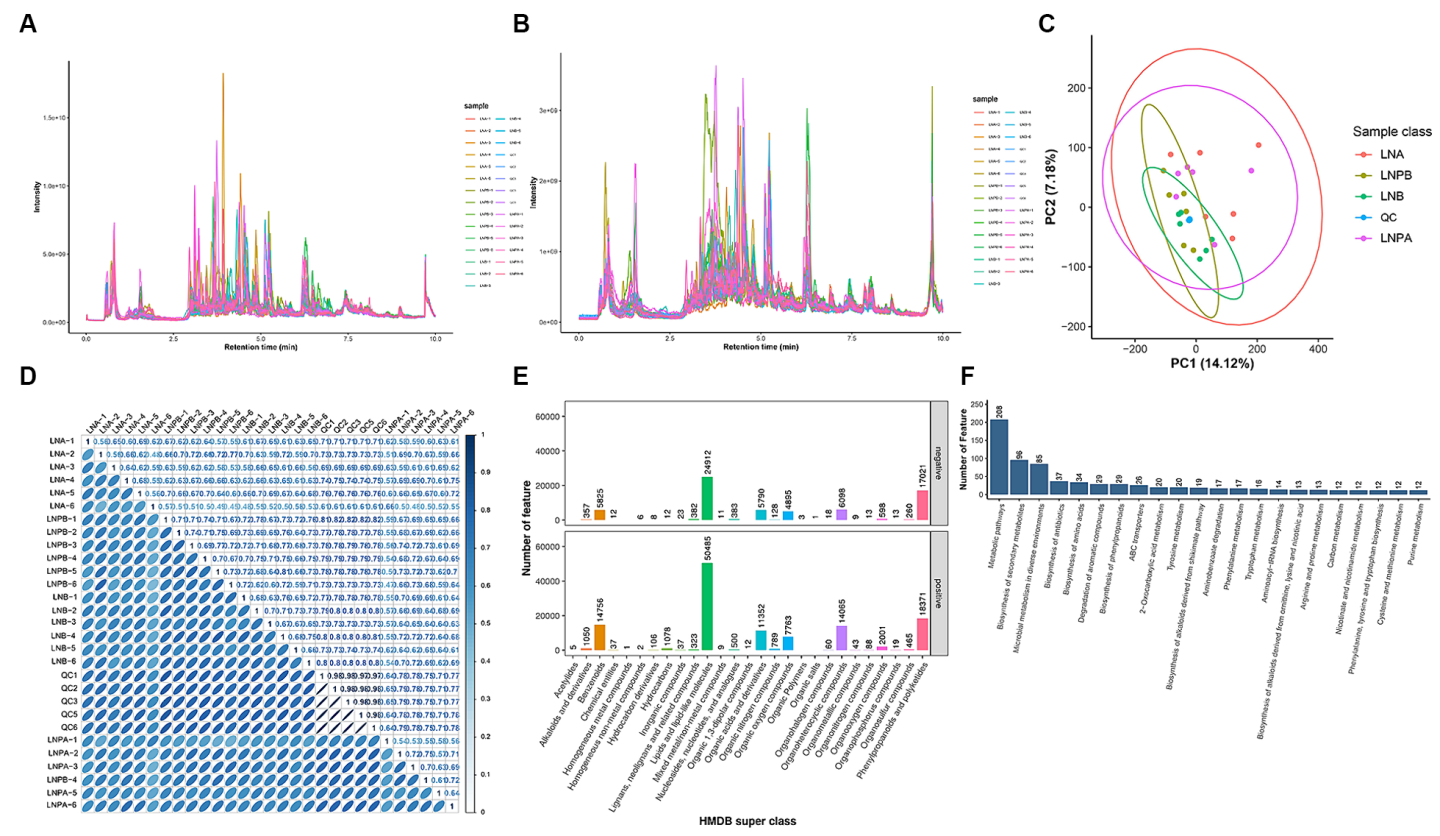
Study of the effect of synbiotics on gut microbiota in lupus nephritis based on non-targeted metabolomic sequencing. QC, blank control. LNA, Lupus group before treatment. LNB, Lupus group after treatment. LNPA, Synbiotic group before treatment. LNPB, Synbiotic group after treatment. **(A)** TIC^a^ chromatogram of metabolites in positive ion mode. **(B)** TIC chromatogram of metabolites in negative ion mode. **(C)** PCA^b^: Abscissa PC1 and ordinate PC2 in the figure represent the scores of the principal components ranked first and second, respectively. **(D)** Correlation coefficient graphs for all samples. **(E)** The HMDB Super class classification diagram, representing super class entries as the abscissa and the number of metabolites corresponding to entries as the ordinate. **(F)** Top 20 KEGG pathways associated with secondary metabolites. ^a^Total Ion Chromatogram; ^b^Principal component analysis.

Firstly, through the quality control of substances extracted by XCMS software, the total ion chromatogram reflected that all metabolites were well separated using LC, indicating efficient sample preparation and high-quality raw data (Figures 6A,B). Principal Component Analysis (PCA) performed on the identified metabolite ions indicated that the grouping settings were reasonable (Figure 6C). Metabolites were identified by metaX software. After relative quantification and data normalization of the identified metabolite ions, the correlation between samples in each group was good (Figure 6D). The candidate substances were annotated by HMDB, KEGG and other databases. Notably, ‘lipids and lipid-like molecules’ were the most abundant (Figure 6E). According to the KEGG classification map, the top 20 KEGG pathways and the largest number of secondary metabolites were associated with the ‘metabolic pathway’ in KEGG (Figure 6F).

**Figure 7:**
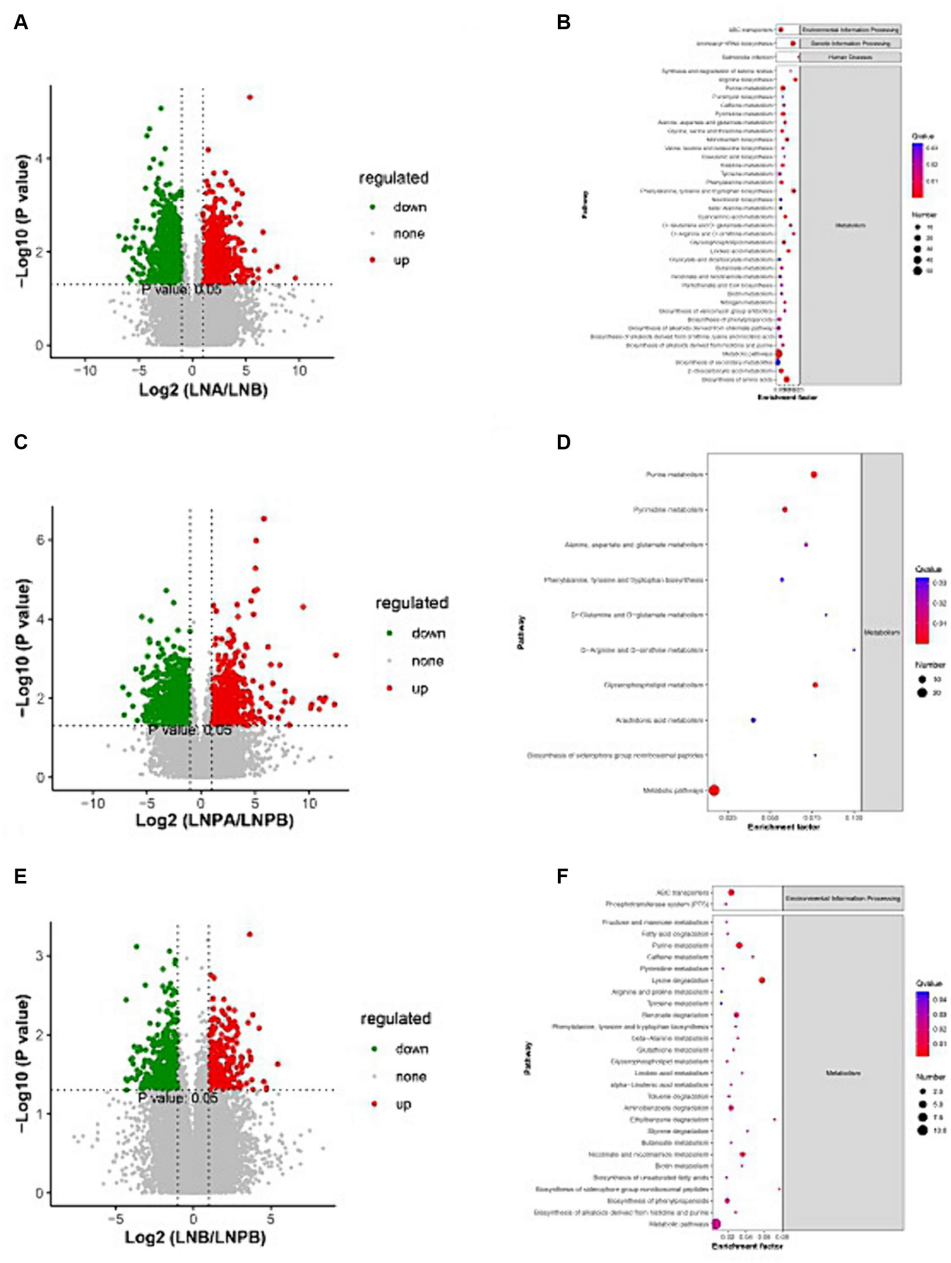
Volcanic map and KEGG enrichment scatter plots of the differential metabolites. **(A)** Volcano map of the lupus group before and after treatment. **(B)** The KEGG enrichment scatter plot of the lupus group before and after treatment. **(C)** Volcano map of the synbiotic group before and after the treatment. **(D)** The KEGG enrichment scatter plot of the synbiotic groups before and after treatment. **(E)** Volcanic maps of the two groups after treatment. **(F)** The KEGG enrichment scatter plot of the two groups after treatment (Volcano diagram: the sizes of the scatter points represent the VIP^b^ values of the PLA-DA model. The greater the dispersity, the greater the VI *p* value. Red represents significantly upregulated metabolites, green represents significantly downregulated metabolites, and grey represents metabolites with no significant differences. KEGG enrichment scatter plot: The rich factor indicates the number of differential genes located in KEGG/total number of genes located in the KEGG database. The smaller the *p*-value, the higher the degree of KEGG enrichment).

The metaX software was used to quantify metabolites and screen differential metabolites. Of the 2,198 annotated metabolites in the lupus group before and after treatment, 852 were upregulated and 1,346 were downregulated. Further, 647 were upregulated and 1,129 were downregulated in the synbiotic group before and after treatment. Moreover, 318 were upregulated and 391 were downregulated in the lupus group after treatment compared with the synbiotic group (Figures 7A,C,E). We conducted a differential analysis and comparison of the identified metabolites. A total of 300 differential metabolites were identified in the lupus group before and after treatment, 77 were identified in the synbiotic group before and after treatment, and 68 were identified when comparing the two groups after treatment, including lipids, lipid-like molecules, organic heterocyclic compounds, organic acids, and their derivatives.

KEGG pathway was enriched by differential metabolites and the enrichment significance was analyzed. Pathway enrichment analysis of the differential metabolites in the lupus group before and after treatment revealed 84 enriched metabolic pathways, 44 of which were significantly differentially enriched (*p* < 0.05), mainly in the biosynthesis of amino acids, aminoacyl-tRNA biosynthesis, and purine metabolism. Further, 55 metabolic pathways were enriched in the synbiotic group before and after treatment, 30 of which were significantly differentially enriched (*p* < 0.05), mainly in purine, glycerophospholipid, and pyrimidine metabolisms. After treatment, 34 metabolic pathways were enriched in the two groups, 29 of which were significantly differentially enriched (*p* < 0.05), mainly in pyrimidine metabolism, lysine degradation, and ABC transport. In addition, a close connection between metabolic pathways and biosynthesis of secondary metabolites in patients with LN was observed (Figures 7B,D,F).

### 3.4 Correlation analysis of differential species, clinical characteristics, and differential metabolites

**Figure 8:**
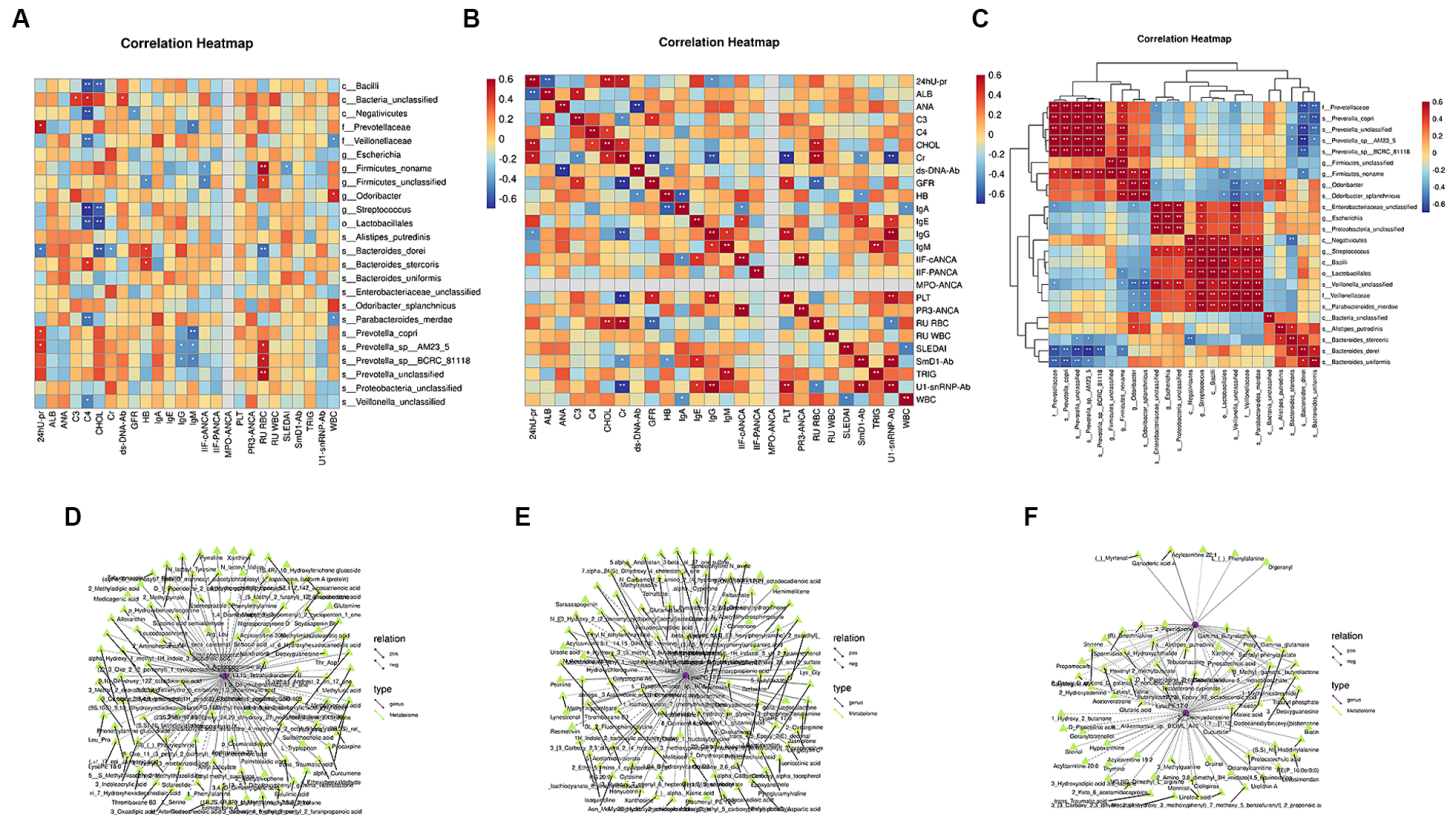
Correlation analysis of differential microbiota with clinical characteristics and differential metabolites. **(A)** Spearman’s correlation analysis between differential microbiota and clinical indicators. **(B)** Spearman’s correlation analysis of clinical indicators. **(C)** Spearman’s correlation analysis of different bacterial communities (Color intensity represents the size of the correlation; red: positive correlation; blue: negative correlation. *p < 0.05; **p < 0.01; ***p < 0.001. Spearman’s correlation coefficient values below 0 indicate blue edges, and coefficient values above 0 indicate red edges). **(D)** Joint analysis network diagram of the differential metabolites and microbiota before and after treatment in the lupus group. **(E)** Joint analysis network diagram of differential metabolites and microbiota before and after treatment in the synbiotic group. **(F)** Joint analysis network diagram of differential metabolites and microbiota in the two groups after treatment (different nodes represent different species or metabolites, and triangles and circles represent species and metabolites, respectively. The solid and dashed lines indicate positive and negative correlations, respectively).

Spearman’s correlation analysis was used to study the correlation between the flora, metabolites, and clinical characteristics. The results revealed an interaction among these factors, and the flora co-established complex biological network regulatory relationships. Notably, 24-h urinary protein was significantly positively correlated with f_Prevotellaceae, s_*Prevotella*, and s_*Prevotella_copri*, and significantly negatively correlated with s_*Bacteroides_dorei*. No significant correlation was identified between flora and ALB. SLEDAI and IIF-cANCA levels were significantly negatively correlated with g_Firmicutes_Noname. Complement C4 significantly negatively correlated with g_*Streptococcus*, o_Lactobacillales, c_Negativicutes, c_Bacilli, s_*Parabacteroides_merdae*, f_Veillonellaceae, and s_*Veillonella*_unclassified. Complement C4 was significantly positively correlated with c_Bacteria_unclassified and s_*Bacteroides_stercoris*. The following four indices were significantly positively correlated only with the differential flora: Complement C3 and c_Bacteria_unclassified. GFR was significantly negatively correlated with c_ Negativicutes. Serum creatinine was significantly negatively correlated with s_*Bacteroides_dorei*. The double-stranded deoxyribonucleic acid antibody (ds-DNA-Ab) was significantly positively correlated with c_Bacteria_unclassified (Figures 8A–C).

KEGG metabolic pathways were combined with metabolomic and metagenomic data. Notably, before and after treatment in the lupus group, *Streptococcus*, s_*Pedosphaera parvula*, s_Gemmatimonadales bacterium, L-serine, 2-aminobenzoic acid, and L-glutamine participated in amino acid biosynthesis. *Streptococcus*, *Chlamydia*, s_*Skermanella*, and S*kermanella*_*stibiiresistens* contain L-serine, L-glutamine, L-phenylalanine, L-tryptophan, threonine, lysine, glutamine, L-glutamic acid, methionine, phenylalanine, arginine, tyrosine, and tryptophan and participate in aminoacyl-tRNA biosynthesis. In the synbiotic group, before and after treatment, *Streptococcus*, s_*Veillonella*_sp., *Butyricoccus*, Rhizobia, adenine, hypoxanthine, adenosine, inosine, guanosine, xanthine, and 2′-deoxyadenosine participated in purine metabolism, whereas butyric acid *Coccus*, s_*Veillonella*_sp., *Rhizobium* and uridine, 5-thymidylic acid, cytosine, and uracil participated in pyrimidine metabolism. After treatment, Rhizobia, xanthine, hypoxanthine, and 2′-deoxyadenosine participated in purine metabolism. *Clostridium* participated in the ABC transporters, together with mannitol, spermidine, and biotin. Therefore, after synbiotic treatment in patients with LN, the enriched metabolites and gut microbes among the groups interacted via multiple metabolic pathways (Figures 8D–F).

## 4 Discussion

Domestic and foreign studies have confirmed different degrees of intestinal flora imbalance in patients with SLE (Sonnenburg and Bäckhed, 2016). LN is one of the most common and serious complications and can potentially cause visceral damage (Anders et al., 2020). To explore the role of intestinal flora in LN, Valiente et al. (2022) colonized NZM2410 mice with segmented filamentous bacteria (SFB) and found that SFB were more active in NZM2410 mice than in NZM2410 mice without SFB. Colonization in mice exacerbates renal disease by promoting renal M2-like macrophage infiltration and gut microbiota dysbiosis. Gut microbes play an important role in maintaining body health, and probiotics, and prebiotics have been investigated as treatment options for these diseases (Mattila et al., 2012; Kalinkovich and Livshits, 2019; Su et al., 2020). A study reported that dietary oatmeal increases fecal microbes, and probiotic supplementation leads to increased host intestinal hydrolytic enzymes and immune function, suggesting that soluble prebiotics supplemented with probiotics may be a more bioactive combination of synbiotics in Sprague–Dawley rats (Kim et al., 2022). Synbiotics colonize lactobacilli to restore barrier function and microbiota and reduce colonic oxidative stress. Notably, combinations of synbiotics may be used in the treatment of alcoholic liver disease (Patel et al., 2022). Synbiotic supplementation can reduce systemic inflammation and SLE disease activity and alter gut microbiota composition and function (Widhani et al., 2022). However, the current research on the therapeutic effects of probiotics, prebiotics, and synbiotics on LN is limited. Therefore, we explored the effects of synbiotics on the conventional treatment of patients with new-onset LN using metagenomic and metabolome sequencing. The gut microbiota and its metabolites are regulated in healthy individuals, and the gut contains approximately 20% *Bacteroides*, 80% Firmicutes, 1% Proteobacteria, and 3% bacteria (Lukiw, 2016). Firmicutes and Bacteroidetes are the main flora involved in the transport and carbohydrate metabolism pathways, respectively (Meehan and Beiko, 2012), and the F/B ratio is an important parameter reflecting the degree of intestinal flora disorder. Firmicutes is the dominant phylum in the gut microbiome (Yacoub et al., 2018), which mainly produces butyrate, an energy source for colonic epithelial cells with anti-inflammatory effects (Chakraborti, 2015). *Bacteroides* was the predominant phylum in our study. Members of the genus *Bacteroides* are potential colonizers of the colon and constitute a major part of the gut bacterial flora (Kim et al., 2017). *Bacteroides* cause inflammation by secreting lipopolysaccharides and toxic proteolytic peptides (Wexler, 2007; Lukiw, 2016). Wexler (2007) reported that the abundance of Firmicutes in patients with LN was significantly lower than that in normal individuals, whereas the abundance of Bacteroidetes was significantly increased, consequently decreasing the F/B ratio. Hevia et al. (2014) reported a lower F/B ratio in patients with SLE than in healthy controls. This study found that the abundance of *Bacteroides* increased and that of Firmicutes decreased in the lupus group after treatment compared with before treatment, which was similar to the findings of Hevia et al. However, the abundance of *Bacteroides* decreased and the abundance of Firmicutes increased after treatment in the synbiotic group compared with before treatment, indicating an imbalance in the intestinal flora of patients with LN. The abundance of Firmicutes increased after synbiotic treatment, suggesting a potential anti-inflammatory effect, consistent with the findings of the study on synbiotics and SLE by Widhani et al. (2022). Further, the abundance of Proteobacteria decreased significantly after treatment in both groups compared with that before treatment, and a statistically significant difference was observed before and after treatment in the synbiotic group. The abundance of Actinobacteria increased significantly after treatment in the synbiotic group compared with that before treatment. All Proteobacteria are gram-negative bacteria, and their outer membranes are mainly composed of 44 lipopolysaccharides. Proteobacteria include many pathogenic bacteria, such as *Escherichia coli, Escherichia, Salmonella, Vibrio, Helicobacter, and Shigella*. Vaziri (2016) reported that Actinomycetes*, Clostridium,* and Proteobacteria are significantly increased in kidney disease, indicating that Proteobacteria may be pathogenic in patients with LN. Actinobacteria are among the oldest bacterial phyla and play important roles in medicine and biotechnology (Ventura et al., 2007; Nouioui et al., 2018). The metabolites of actinomycetes are rich in variety, structurally complex, and exhibit antitumour, antibacterial, antiviral, and antituberculosis activities. Seventy percent of the discovered antibiotic-active substances are derived from actinomycetes (Kothe, 2018). Li et al. (2019) found that Actinobacteria increased and Tenericutes decreased in stool samples from patients with SLE. We found that the abundance of Proteobacteria decreased and that of Actinobacteria increased after treatment with synbiotics, indicating that synbiotics affected both pathogenic and beneficial bacteria. At the genus level, it mainly affected *Bacteroides, Prevotella, Faecalibacterium,* and *Parabacteroides*. The genus *Prevotella* includes more than 50 characteristic species (Tett et al., 2021) and their members are associated with various diseases, including inflammatory autoimmune diseases (Scher et al., 2013), opportunistic infections (Teanpaisan et al., 1996; Baumgartner et al., 1999; Bein et al., 2003; Zhao-Fleming et al., 2018), bacterial vaginosis (Si et al., 2017), and oral biofilm formation disorders (Teles et al., 2012; Larsen, 2017). Notably, in the present study, the abundance of *Prevotella* in the lupus group decreased after treatment compared with that before treatment, and its expression was upregulated, which may be involved in the occurrence and development of LN diseases.

Further, α diversity analysis revealed no significant differences in the intestinal microbial richness or diversity index of patients with LN between the two groups before and after treatment. Another study, using the stool samples of the patients, revealed that the bacterial richness and diversity decreased in the patients with SLE compared with the healthy control group (Liu et al., 2021). The description of intestinal microbial diversity in our experimental results was inconsistent with the above reports. β analysis showed that the samples before and after treatment in the two groups were generally far apart, along with the differences in the community structure. This may be due to the intestinal regulatory effect of synbiotics in patients with lupus. However, other reasons for these differences, such as the inclusion criteria for the experiment and the impact of the number of inclusions on the results, also need to be considered. Subsequently, future studies with more data and a precise design are required to confirm species abundance and diversity.

LEfSe analysis revealed 15 biomarkers in the lupus group before and after treatment. Prevotellaceae exhibited the highest score (LDA = 4.90), and 35 biomarkers were identified before and after synbiotic treatment. Further, *Bacteroides_*unclassified exhibited the highest score (LDA = 4.84). The results of the ROC analysis showed that Negativicutes (AUC = 0.9722) was the best predictor in the lupus group before and after treatment. Negativicutes are gram-negative bacteria with two cell membranes; however, they are phylogenetically a collateral branch of gram-positive Firmicutes, containing only one membrane, classified within three orders (Yutin and Galperin, 2013). Sporobacteriaceae strain FL31 is a new lactic acid-fermenting bacterium belonging to the class Negativicutes (Aoyagi et al., 2019). The draft genome of the FL31 strain is associated with lactic acid fermentation, which produces propionate and acetate via the Wood–Werkman pathway (Gonzalez-Garcia et al., 2017). The best predictor in the synbiotic group, before and after treatment, was Enterobacteriaceae*_*unclassified (AUC = 0.9722). Dong et al. (2020) found that membranous nephropathy is characterized by immune disorders and is associated with intestinal malnutrition. The abundance of Enterobacteriaceae_unclassified was higher in the membranous nephropathy group than that in the healthy control group. This study also found that the abundance of Enterobacteriaceae_unclassified before treatment in the lupus group was significantly higher than that after treatment and that the abundance of Enterobacteriaceae_unclassified after treatment in the synbiotic group was lower than that in the lupus group, suggesting that Enterobacteriaceae_unclassified is also involved in the occurrence and development of LN. The composition ratio of the intestinal flora in patients with LN and dysbiosis is involved in LN pathogenesis.

KEGG enrichment analysis revealed that, compared to before treatment, the expression of genes of intestinal flora in the two groups after treatment was upregulated and mainly enriched in the synbiotic group. Compared with the synbiotic group, the intestinal microbial metabolic function was more vigorous and the expression level was higher, indicating that the addition of synbiotics regulated the intestinal microbiota and enhanced its metabolic function. Combining non-targeted metabolomics and macro-combined genomic analysis revealed that a variety of differential metabolites, including amino acid biosynthesis, aminoacyl-tRNA biosynthesis, purine metabolism, glycerophospholipid metabolism, pyrimidine metabolism, lysine degradation, ABC transport, and other metabolic pathways, may be involved in patients with LN. The mechanism of metabolic function changes after synbiotic administration, and the enriched metabolites and gut microbes interact via multiple metabolic pathways.

Finally, statistical analyses were performed to explore changes in clinical biochemical indicators in patients with LN after synbiotics administration. According to current domestic literature reports, the male-to-female prevalence ratio of patients with LN is 1:5.5–9.7 (Stefanidou et al., 2013). Diet, age, gender, race, and BMI may affect the composition of the flora (Bolnick et al., 2014; Deschasaux et al., 2018; He et al., 2018) and hence, they were matched in this study. As the patients included in this study were all women from western Guangxi, the changes in gut microbes in patients with LN after synbiotics administration based on sex could not be compared. Men with SLE have a higher mean age of onset (Renau and Isenberg, 2012; Tan et al., 2012), more severe organ damage, and a higher incidence of LN than women with SLE (Gui et al., 2022). Studies have also shown a statistically significant difference in the age of onset between men and women, which may be related to differences in region, race, case selection, and sample size (Deschasaux et al., 2018). The results of this study showed that after treatment in the lupus and synbiotic groups, the values of SLEDAI, 24-h urine protein, WBC count, and other indicators decreased, whereas the values of ALB, HB, GFR, and other indicators increased. PLT and ALB differ significantly after treatment in the synbiotic group compared with before treatment. SLEDAI, 24-h urine protein, PLT and ALB differ significantly after treatment in the synbiotic group compared with before treatment. The 24-h urine protein was significantly positively correlated with f__Prevotellaceae, s__Prevotella, and s__Prevotella_copri. We know that 24-h urine protein is related to the severity of LN. As mentioned above, at the genus level, we found that the abundance of *Prevotella* decreased after treatment in the lupus group compared with that before treatment, which once again indicates that *Prevotella* may be involved in the occurrence and development of LN. We found that after treatment with synbiotics, the abundance of Bacteroidetes decreased and the abundance of Firmicutes increased. Relevant studies have found that the content of Firmicutes in LN patients was significantly lower than that in normal people, while the content of Bacteroidetes was significantly increased (Hevia et al., 2014). In this study, Spearman correlation analysis showed that 24-h urine protein was significantly negatively correlated with s__Bacteroides_dorei. No significantly related flora was found for ALB. SLEDAI was significantly negatively correlated with g__Firmicutes_noname. We found that SLEDAI decreased after treatment with synbiotics, with a statistically significant difference compared with that before treatment. The abundance of Firmicutes increased after treatment with synbiotics, indicating that the abundance of harmful flora in the intestine of LN patients decreased after treatment with synbiotics, and the abundance of beneficial flora increased. Spearman’s correlation analysis between the characteristics of the intestinal flora and clinical parameters revealed the interaction between them, and flora coexisted and showed complex biological network regulatory relationships. The deposition of various autoantibodies plays a key role in the onset, progression, and severity of LN and is of great value in its diagnosis, treatment, and prognosis (Oelzner et al., 2003; van der Vlag and Berden, 2011). Anti-ds-DNA and ANA-specific antibodies are specific to systemic autoimmune diseases (Anis et al., 2023), and ds-DNA-Ab was significantly positively correlated with c_Bacteria_unclassified in our study. SLEDAI is a commonly used disease activity scoring tool in clinical practice. Based on the SLEDAI score, the glycosylation of anti-dsDNA IgG and total IgG subclasses was analyzed to evaluate their correlation with disease activity. We found that anti-dsDNA IgG1 fucosylation of serotonin correlated best with SLE disease activity (Han et al., 2022). A study exploring the safety and efficacy of fecal microbiota transplantation (FMT) in the treatment of patients with SLE reported a significant reduction in SLEDAI-6 K scores and serum anti-dsDNA antibody levels, confirming that FMT alters the gut microbiome and its metabolic profile and may be a feasible, safe, and potentially effective treatment for patients with SLE (Huang et al., 2022). We found that SLEDAI was significantly negatively correlated with g_Firmicutes_noname, indicating that Firmicutes were involved in the regulation of the gut microbiota in LN after synbiotic treatment. The results of this study indicated no significant differences in immune-related indicators between the two groups before and after treatment. Correlation analysis revealed that complement C3 was significantly positively correlated with c_Bacteria_unclassified and IgG was significantly negatively correlated with s_*Prevotella*. Proteinuria is a common clinical manifestation of LN. Notably, the cytokines IL-1RA and LIF are strongly positively correlated with 24-h urine protein levels (Zhang et al., 2022). Further, ROC analysis revealed that IL-1RA has a good diagnostic value, and IL-10 and LIF levels can be used to differentiate between active and inactive SLE (Zhang et al., 2022). We found that 24-h urine protein levels were significantly and positively correlated with f_Prevotellaceae, s_*Prevotella*, and s_ *Prevotella_copri*.

In summary, our study revealed specific changes in gut microbiota and metabolite profiles after synbiotics administration in patients with LN. Through integrated data analysis, we also identified the intestinal flora that was closely related to the clinical indicators of LN and the KEGG pathway, which may be involved in regulating changes in the intestinal flora and metabolites after synbiotic treatment. Our study provides information on improvements in patients with LN, provides clues regarding their immune status, and sheds light on the potential of microecological therapies targeting LN. This study has limitations. On the one hand, the sample size is relatively small, and more precise research is needed to understand the relevant mechanisms between the gut microbiota and metabolic product profiles. On the other hand, targeted metabolic mass spectrometry analysis is needed to further clarify the main metabolites and metabolic pathways that affect the immune phenotype of LN. Relevant studies have shown that kynurenine is the primary predictor of N-ace tyrosine (NAC) effect in SLE, The pentose phase pathway (PPP)-connected and NAC-responsive accumulation of kynurenine and its stimulation of mTOR are identified as novel metabolic checkpoints in lupus pathogenesis (Perl et al., 2015). The interplay of gut microbial dysbiosis, tryptophan metabolism and host genetic susceptibility suggested that aberrant tryptophan metabolism in lupus-susceptible mice could be one of the mechanisms contributing to autoimmune activation in this disease (Choi et al., 2020). Microbiota-mediated skewing of tryptophan catabolism modulates CD4^+^ T cells in lupus-prone mice (Brown et al., 2022). Once again, we need to further explore the interaction mechanisms between microorganisms, metabolites, metabolic pathways, and LN after adding synbiotics from aspects such as zoology and cytology.

## Data availability statement

The datasets presented in this study can be found in online repositories. The names of the repository/repositories and accession number(s) can be found at: NCBI – PRJNA977213.

## Ethics statement

The studies involving humans were approved by the Ethics Committee of the Affiliated Hospital of Youjiang Medical University for Nationalities (YYFY-LL-2020-031). The studies were conducted in accordance with the local legislation and institutional requirements. The participants provided their written informed consent to participate in this study.

## Author contributions

QZ: Formal analysis, Methodology, Writing – original draft. JC: Formal analysis, Writing – original draft, Data curation. SL: Formal analysis, Writing – original draft, Methodology. SW: Investigation, Project administration, Writing – review & editing. QW: Investigation, Project administration, Writing – review & editing. YY: Project administration, Writing – review & editing, Funding acquisition.
